# Smoking and Medication During Pregnancy Predict Repeated Unintentional Injuries in Early Childhood But Not Single Unintentional Injuries

**DOI:** 10.1007/s11121-012-0304-3

**Published:** 2012-12-05

**Authors:** Marianne Junger, Christa Japel, Sylvana Coté, Qian Xu, Michel Boivin, Richard E. Tremblay

**Affiliations:** 1 Institute for Innovation and Governance Studies, University of Twente, P.O. Box 217, 7500 AE Enschede, The Netherlands; 2 University of Québec at Montreal, Montréal, Canada; 3 University of Montréal, Montréal, Canada; 4 Laval University, Québec City, Canada; 5 School of Public Health, Physiotherapy and Population Science, University College Dublin, Dublin, Ireland; 6 Departments of Paediatrics, Psychiatry and Psychology, University of Montreal, Montreal, Canada; 7 INSERM Unit 669, Paris, France

**Keywords:** Early prevention, Unintentional injury, Prenatal smoking, Smoking, Prenatal substance use

## Abstract

This study investigates prospectively the development of single and repeated unintentional injuries from birth to 42 months in a random population sample of new-born children in Quebec (Canada) (*N* = 1,770). The outcome measures are single unintentional injuries (SUI) and repeated unintentional injuries (RUI). Results showed that the risk factors for SUI differed from the risk factors for RUI. SUI was predicted by mother’s antisocial behavior during high school (*OR* = 1.72) and mother’s age at first birth (*OR* = 1.82) with children from older mothers at higher likelihood of SUI. Also, boys (*OR* = 1.36) and hyperactive children (*OR* = 1.06) were at increased risk of SUI. RUI was predicted by maternal smoking during pregnancy (*OR* = 1.68), medication on prescription (*OR* = 1.53) and medication without prescription (*OR =* 1.54). Boys (*OR =* 2.01), children with a difficult temperament (*OR =* 1.13) and those with single mothers had higher rates of RUI (*OR =* 2.05). Maternal perception of impact (*OR =* 1.15) and maternal feelings of self-efficacy (*OR =* 0.87; marginally significant) were also associated with RUI. These results show that maternal and child risk factors identified during pregnancy and just after birth can predict SUI as well as RUI in early childhood. However, the only common risk factor for SUI and RUI is the child’s sex, with boys being at higher risk than girls. Implications of these findings and suggestions for prevention are discussed.

Unintentional injuries are a major health problem (Segui-Gomez and MacKenzie 2003), a huge economic burden (Glied 1999), and a major cause of child mortality and morbidity (Valent et al. 2004). A clearer picture of the risk factors may lead to develop and improve preventive efforts. The objective of this study is to trace the development of repeated unintentional injuries (RUI), defined as two or more injuries, during the first 4 years after birth. This study is the first to describe the development of RUI over the first 4 years of life within a large birth cohort. Second, it examines whether prenatal risk factors and factors measured during the first 17 months are independent predictors of children’s unintentional injuries over that period of time. Risk factors for RUI will be compared with risk factors for single unintentional injuries (SUI). The predictors of RUI (or ‘injury recidivism’) are not necessarily the same as those of SUI (Russell 1998).

This study brings together two different fields that have not been well connected until now: 1) research investigating risk factors for unintentional injuries during childhood, and 2) predictive studies of child development using mother’s prenatal characteristics within a life-course approach.

Research on risk factors has shown that the postnatal psychosocial environment of the child and the child’s characteristics are related to the risk for unintentional injuries. Children at risk for unintentional injuries have relatively often mothers who are young (Ekeus et al. 2004), poorly educated (Soubhi et al. 2002), single (Ekeus et al. 2004), depressed (Schwebel and Brezausek 2008), have a history of antisocial behavior (Hjern and Bremberg 2002) and live in underprivileged neighborhoods (Durkin et al. 1994).

Several aspects of parenting are also associated with children’s injuries. More specifically, poor quality of supervision by parents increases the likelihood of unintentional injuries in children (Morrongiello 2005).

Among children’s characteristics three factors have been documented relatively well in the literature: sex, hyperactivity and antisocial behavior. Boys are at higher risk for unintentional injuries than girls (Owens 2002). Behaviors related to hyperactivity and/or an attention deficit are also risk factors for unintentional injuries (Barkley et al. 1993; Bijur and Stewart-Brown 1986; Langley et al. 1983). In addition, child conduct problems and antisocial behavior has been found to increase the incidence of intentional injuries (Rowe et al. 2004; Teplin et al. 2005; Van Aken et al. 2007). Lastly, low BMI is associated with injuries (Thompson et al. 2011).

Research on maternal predictors of child development from prenatal characteristics has focused on how these affect important negative outcomes among their children. Much attention has been paid to maternal substance use and its effect on child outcomes. Various forms of substance use are relatively common during pregnancy (McKenna and McIntyre 2006). There is ample evidence from animal and humans studies that many drugs cross the placenta (Syme et al. 2004) and have organic and behavioral teratogenic effects on the development of the fetus (Mattson et al. 2001; National Scientific Council on The Developing Child 2006). Numerous studies documented a relationship between prenatal parental tobacco smoking and chronic physical aggression, conduct disorders and attention deficit/hyperactivity disorder (ADHD) (Huijbregts et al. 2008; Linnet et al. 2003). One study established a link between substance use (for the definition of substance use: see the Discussion) during pregnancy and childhood unintentional injuries during the first 24 months: 13.1 % of the children in the substance abuse group were subject to injuries vs 6.4 % in a comparison group (Ellwood et al. 1993).

We follow a life course approach in the present longitudinal study. The life course approach to health and disease integrates social and biological developmental pathways. It emphasizes timing of risk factors, uses the concepts of critical or sensitive periods, and studies the intergenerational pathways of transmission of risk and disease (Ben-Shlomo and Kuh 2002; Halfon and Hochstein 2002). Wadsworth (1998) hypothesizes that poor health in the first years of life is “*a reflection of “biological programming”* (quotes in the original) *in utero, that is processes that are “hardwired” into the child from birth.*”(p. 47) Wadsworth (Wadsworth) proposes a chain of events in which biological factors interact with the environment to produce health outcomes that include risk of injuries.

The present study examines how many unintentional injuries occurred in this sample and which risk factors are associated with their occurrence. It investigates whether various forms of maternal substance use, maternal characteristics, maternal perceptions and behaviors, and child characteristics are associated with SUI and RUI in children. It uses a life course perspective by including early predictors and by investigating their effect over the first years of life. Potential risk factors predicting RUI and SUI are ordered as they occur and are measured concurrently to the development of the child. More precisely, the first cluster of risk factors to predict RUI and SUI comprises characteristics of the mother, namely her educational level, her antisocial behavior during high school and her age at the birth of her first child and prenatal use of various substances. The second set of risk factors contains information obtained at the child’s birth such as sex, and whether the mother is a single parent at the time of birth. In the third step, we include information on family income, maternal depression and child temperament, measured during the first months of the child’s life. Lastly, measured when the child is 17 months old, children’s hyperactivity behaviors, body mass index and maternal perceptions on socialization are included to predict RUI and SUI.

As mentioned above, this developmental sequence matches more closely to the ‘chain of risk’ in human development (Shonkoff and Phillips 2000) and it corresponds to a more developmental approach in public health (Ben-Shlomo and Kuh 2002; Halfon and Hochstein 2002). To our knowledge, no study has included a random population sample and examined prospectively the development of unintentional injuries during the first 4 years of life.

## Method

### Participants

The Quebec Longitudinal Study of Child Development (QLSCD) follows a representative sample (Jetté and Des Groseilliers 2000) of children born in the province of Québec, Canada, between October 1st 1997 and July 31st 1998. The target population of the survey are the babies, singleton births only (twins were excluded) who were 59 or 60 weeks of gestational age, born to mothers residing in Québec, excluding those living in the following regions: Northern Québec, Cree “territory,” Inuit “territory,” and Indian reserves. According to the Master Birth Register for 1997–1998, these exclusions represented 2.1 % of all live births to mothers residing in Québec. Babies were also excluded if the duration of the pregnancy was not indicated in the birth record (1.3 %). At this step in the process, the target population represented approximately 96.6 % of the total population.

Children were selected at birth from the Master Birth Registry of the Health and Social Services Ministry and the sample was constituted through a stratified three-stage sampling procedure (Jetté and Des Groseilliers 2000). Of the initial 2,817 households, 689 households refused to participate, and 8 were ineligible. This resulted in a final sample of 2,120 families for the first wave. From this sample of 2,120 families, 135 left the study over the next 2 years. Of these 135 families 18 moved out of Quebec, 13 could not be found, 1 family had lost their child, and the rest refused or could not participate. This resulted in a sample of 1,985 families when the babies were age 30 months (Institut de la Statistique du Québec 2000, 2002). When the children were 42 months old, 1,926 families remained in the study (wave 4) and provided valid information on injuries. Reasons for this further attrition were not available. Complete data on all the measures included in the present study were available for 1,770 families. Written informed consent was obtained from the parents who participated in the study.

Starting in 1998, when the children were 5 months old, annual interviews were carried out with “the person most knowledgeable about the child.” This was the person taking care of the baby, when he/she was 5 months old and interviewers attempted to keep that person as the person who was interviewed in each wave. In the first wave, in 99.6 % of all families, questions were answered by the biological mother. This percentage decreased slightly to 99.4 %, 98.7 % and 98.4 % for the next waves. In the first wave, in eight families (0.4 %) the biological fathers answered the questions, and this percentage increased to 0.6 %, 1.1 % and 1.4 % in the next waves. In the second, third, and fourth wave, one stepparent answered questions (sex unknown), and in the third and fourth wave, one grandparent and on two occasions a person who was taking care of the child was the source of information (each time: sex unknown). The study was approved by the Ethics Committee of the Quebec institute of statistics (Institut de la Statistique du Québec).

### Measures

#### Unintentional Injuries

At age of 5, 17, 30 and 42 months mothers were asked: *‘The following questions refer to injuries, such as a broken bone, bad cut or burn, head injury, poisoning, or a sprained ankle, which occurred in the past 12 months, and were serious enough to require medical attention by a doctor, nurse, or dentist. In the past 12 months was your child injured?*’. Respondents were asked to name the number of injuries the child had sustained. The frequency measures from age 5, 17, 30 and 42 months were summed. For SUI the children were divided into two categories: 0 (no injury) and 1 (one). We refer to the children with one injury as the group with SUI. For RUI two categories were used: children with no or only one injury (0) and those with two or more (1). We refer to the children with two or more as the group with RUI. A validation study reported that parents are a reasonably valid source of information on the unintentional injuries suffered by their children but are better in reporting relatively serious injuries (Cummings et al. 2005; Harel et al. 1994).

The risk factors are presented below in order of entry in the logistic regression analysis. The first interview, when the children were 5 months old, provided information on all the risk factors used in steps 1, 2 and 3. Information for step 4 comes from the assessment at age 17 months.

Mothers’ educational level was measured in four categories: no high school diploma (16.4 %), only high school diploma (25.4 %), more than high school but less than university (29.8) and university diploma (28.4 %).

Mothers’ antisocial behavior was assessed by asking whether, before the end of high school, she had started more than one fight, stolen more than once, been involved with youth protection or the police because of misbehavior, skipped school more than twice in 1 year, or ran away from home overnight (Zoccolillo 2000). Responses were summed and formed three categories: no antisocial behavior (45.1 %), one form of antisocial behavior (36.5 %) and two or more forms of antisocial behavior (18.4 %). This scale has been used in previous research and was found to predict physical aggression in children (Zoccolillo 2000).

For mother’s age at first birth we used the categorization ‘lowest to 15’, ‘16–19’, ‘20 to 23’ and ‘24 and higher’ (Phipps and Sowers 2002). As only six mothers were age 14 or 15, this category was collapsed with ‘16–19’.

Mother’s substance use was assessed when several questions were asked about her health behavior during pregnancy. For each pregnancy trimester, mothers were asked how many cigarettes they smoked daily, whether during pregnancy they used alcohol, medication on prescription, medication without prescription or illicit substances.

Smoking was coded as 0 (no smoking: 74.8 %) or 1 (smoking: 25.2 %). Preliminary analyses showed that there was no dose-effect relationship between mothers’ smoking and children’s RUI. The difference was between mothers who did not smoke and mothers who smoked. Among mothers who did not smoke, 4.4 % of the children had RUI. Among mothers who smoked, this figure varied between 7.9 % and 8 %. The largest group of mothers who smoked was the group who reported to smoke between 6 and 10 cigarettes a day (*N* = 164). As a result, this variable was entered in the regression as a dichotomous variable.

To measure alcohol use the question was asked ‘how many glasses do you usually drink?’. Answers were coded as none (62.6 %), one or two (36.8 %) and three or more (0.6 %).

For medication on prescription, medication without prescription and illicit substances no further details were asked about what type of medication or substance was used. These three variables were coded 0 (the risk behavior was not mentioned) or 1 (the risk behavior was mentioned). Medication on prescription was used by 60 % of the mothers, 61.2 % reported the use of medication without prescription and 1.2 % of mothers used illicit substances during pregnancy.

Sex was coded 0, for female (50.3 %) and 1 for male (49.7 %). Family status at the child’s birth was coded 0 when both parents were present (94.2 %), and 1 when the mother was single (5.8 %).

Annual income was measured and coded in nine categories (see Table 1).

**Table 1:** Sample characteristics, frequencies, and bivariate relationships with single unintentional injuries (SUI) (*N* = 1,678) and repeated unintentional injuries (RUI) (*N* = 1,770)

		*N*	SUI	*N*	RUI
Step 1					
Mother’s educational level	No high school diploma	279	20.4	291	4.1
	Only high school diploma	427	20.4	449	4.9
	More than high school but less than university	492	22.2	528	6.8
	University diploma	481	20.4	502	4.2
Mother’s antisocial behavior (prior to end of high school) **	None	763	18.1	798	4.4
	One	613	21.2	646	5.1
	Two or more	303	27.4	326	7.1
Mother’s age at first birth	20 or younger	151	14.6	223	3.8
	20–23	387	22.0	512	6.3
	24 and older	1141	21.4	1426	4.9
Mother smoked during pregnancy †	No	1265	20.7	1324	4.5
	Yes	414	21.5	446	7.2
Mother drank alcohol during pregnancy	none	1053	20.9	1108	5.0
	One or two glasses	615	21.1	651	5.5
	Three glasses or more	11	9.1	11	0.0
Mother took medication on prescription †	No	1017	22.2	1062	4.2
	Yes	662	18.9	708	6.5
Mother took medication without prescription †	No	1037	20.7	1084	4.3
	Yes	642	21.2	686	6.4
Mother used illicit substance	No	1659	21.0	1748	5.1
	Yes	20	15.0	22	9.1
Step 2					
Child’s sex ** ††	Female	855	18.1	886	3.5
	Male	824	23.8	884	6.8
Mother single at child’s birth †	No	1586	20.9	1667	4.9
	Yes	93	20.4	103	9.7
STEP 3					
Annual income	Less than 10000 CAD	43	20.9	45	4.4
	10,000–14,999 CAD	109	22.0	117	6.8
	15,000–19,999CAD	74	13.5	81	8.6
	20,000–29,999 CAD	212	24.1	223	4.9
	30,000–39,999 CAD	285	21.9	284	6.7
	40,000–49,999 CAD	242	16.1	253	4.3
	50,000–59,999 CAD	200	20.0	214	6.5
	60,000–79,999 CAD	282	24.8	296	4.7
	More than 80,000 CAD	252	19.8	257	1.9
BMI	Between -1 SD and 1 SD	1266	21.5	1337	5.3
	Lower than -1 SD	82	18.3	88	6.8
	Between 1 SD and 2 SD	89	19.1	93	4.3
	More than 2 SD	38	10.5	38	0
	Missing	204	21.1	214	4.7

*SUI*: *Significant: *p* < 0.05; **Significant: *p* < 0.01

RUI: †Significant: *p* < 0.05; ††Significant: *p* < 0.01

In order to measure maternal depressive symptoms mothers were administered an abridged version of the CES-D scale which was developed by Radloff (1977). This instrument contains 12 items such as “I did not feel like eating; my appetite was poor”; and “My sleep was restless.” Mothers were asked to indicate whether they experienced these symptoms on 1 or 2 days, 3 or 4 days, or 5 to 7 days during the previous week. Higher scores indicated higher levels of depressive symptoms. The average Cronbach α coefficient was 0.82.

#### Child Difficult Temperament and Behavior

When the baby was 5 months old, mothers were asked the degree of difficulty their child presented to them. Using the Infant Characteristics Questionnaire (ICQ; Bates et al. 1979), parents were asked to indicate on a scale of 1 (easy) to 7 (difficult) how they perceived the behavior of their baby compared to an “average” or “typical” baby. The seven items comprising the difficult temperament scale included questions such as “How many times per day, on average, does he/she get fussy and irritable - for either short or long periods of time?” Please rate the overall degree of difficulty he/she would present for the average parent. Cronbach’s α was 0.73.

#### Hyperactive Behaviors

Starting at age 17 months, parents were asked to report if their children never, sometimes or often manifested behaviors of hyperactivity. Seven items were used to assess these dimensions originating from the Social Behavior Questionnaire (Tremblay et al. 1994). Items to assess hyperactivity at age 17 months included: can’t sit still, is agitated, easily distracted, fidgets, is impulsive, has difficulty waiting for his/her turn and can’t stay still. Cronbach’s α was 0.74.

#### Parenting Perceptions and Behaviors

The Parental Perceptions and Behaviors Regarding the Infant Scale (PPBS; Boivin et al. 2000) was developed for the Quebec Longitudinal Study of Child Development. It comprises six dimensions on which parents rate their perceptions and behaviors using a response scale ranging from 0 (not at all what I feel or do) to 10 (exactly what I feel or do). Cronbach’s α’s for the different scales ranged from 0.68 to 0.78. Three dimensions of the scale were used in the analyses:

- Maternal feeling of self-efficacy: This subscale measures parental perceptions with regard to the ability to accomplish tasks related to fulfilling the role of parent. The 6-item scale contains statements and questions such as “I feel that I am very good at keeping my baby amused”; “I feel that I am very good at calming my baby down when he/she is upset, fussy or crying” and “In general, do you think you are a good mother/a good father?.”
- Maternal perception of lack of impact. This 5-item scale measures the mother’s evaluation of the effect of her behavior on the development of her child. Items include statements such as “My behavior has little effect on the personal development of my baby” and “My behavior has little effect on how my baby will interact with others in the future.”
- Mother’s tendency to coercion. Seven items of the PPBS address the mother’s proclivity to respond in a hostile and restrictive manner to difficult behaviors in the baby, revealing a lack of sensitivity to his/her needs and moods. This tendency is measured through items such as “I have been angry with my baby when he/she was particularly fussy” and “I have shaken my baby when he/she was particularly fussy.”

#### Body Mass Index

Obesity was computed as ${\text{BMI}} = {\text{Weight}}\left( {\text{kg}} \right)/\left( {{\text{height}}\left( {\text{m}} \right)} \right) * {2}$. Scores were calculated based on the deviation from the mean (mean BMI = 18,3, SD = 3.5; see Table 1). This variable was coded as having four categories. A “missing” category was included to avoid missing too many cases in the analysis.

### Analysis

First, cross tables were computed between SUI, RUI, and the prenatal risk factors. For continuous variables differences of means were examined using analyses of variance. Logistic regression analysis with a stepwise backward procedure was used to study the relationship between the risk factors and SUI and RUI. In step 1, mother’s educational level, antisocial behavior, age at first birth, and substance use during pregnancy (smoking, alcohol use, medication on prescription, medication without prescription and illicit substances) were entered. Step 2 comprised the child’s gender and family status. Step 3 included annual income, maternal depressive symptoms and child difficult temperament. Finally, step 4 consisted of the child’s hyperactive behaviors and BMI, and parental perceptions and behaviors such as maternal feelings of self-efficacy, maternal perception of lack of impact, and mother’s tendency to coercion.

As mentioned above, 1926 families were still in the study at wave 4 (when the child was 42 months old). An attrition analysis was performed by comparing the 1770 families included in the analyses with the 350 for which only information from the first data collection was available. In total, 16 differences were investigated and 7 were found to differ between the families that remained in the study and those that could not be retained. This analysis (tables available from the first author) revealed that the families that had dropped out differed in a number of ways. Compared to mother who stayed in the study, those who did not continue had relatively lower educational levels (no high school diploma: 17 % vs 27 %, respectively), more often had their first child at a younger age (20 or younger: 9 % vs 20 %, respectively), were more likely to be single (63 % vs 74 %, respectively) and had a lower income at the time of the child’s birth. Mothers who left the study drank alcohol less often during pregnancy (17 % vs 6 %, respectively) and slightly more often had a male child (56 % vs 50 %, respectively). They also reported more depressive symptoms in comparison with mothers who stayed in the study (1.6 vs 1.4, respectively). However, there were no significant differences on other characteristics such as smoking during pregnancy, medication use on or without prescription, illicit substance use, and antisocial behavior during high school. Also, there were no significant differences with respect to their children’s difficult temperament at the age of 5 months.

Lastly, it was possible to investigate attrition with respect to unintentional injuries at the age of 5 months. Sustaining unintentional injuries at that age was not related to leaving or remaining in the study as the same number of children that had suffered at least one injury (1.5 %) was equal in the families that dropped out and those that did not.

Nonetheless, it seems that for six of the seven risk factors, the families that did not stay in the study had higher risks scores. Although these differences are small and the total attrition is limited (*N* = 350), this bias towards losing the high-risk group for the study could lead to a slight underestimation of the effects of the predictors of injuries.

## Results

Cumulative rates for single unintentional injuries among boys increase gradually from 1.3 % at 5 months, to 7.4 % at 17 months, 15.4 % at 30 months and 23.8 % at 42 months; for girls, these figures are 0.7 %, 5.7 %, 12.4 % and 18.1 %, respectively. At 5, 17 and 30 months, the differences between boys and girls are nonsignificant, at 42 month the difference between boys and girls is statistically significant (*p* < 0.05).

For repeated unintentional injuries figure are lower. At 5 months no child sustained two injuries. Cumulative rates for boys increased from 0.3 % at 17 months, 2.9 % at 30 months and 6.6 % at 42 months; for girls, these figures are 1.1 % at 17 months, 2.5 % at 30 months and 3.6 % at 42 months, respectively. At 30 months, the difference between boys and girls is nonsignificant, at 17 and 42 month the differences between boys and girls are statistically significant (*p* < 0.05). By the age of 42 months, a total of 439 children had sustained injuries.

Close to one in five of the children (19.8 %) had one unintentional injury and 5.1 % (91 children) had two or more injuries. This second group was responsible for 38 % of all injuries.

Most RUI resulted from falls (once: 41.8 %, twice 28.6 %, three times 3.3 %) or from ‘other causes’ (once: 34.1 %, twice: 12.1 %). Three children were burned by hot liquids and three burned themselves with fire, only one child was injured as a car passenger, one as cyclist, and one during exercise. There were no significant differences between males and females except for burns from hot liquids, which happened only to females (*p* < 0.05).

Table 1 shows the results from the cross-tabular analysis of the risk factors for SUI and RUI. The findings show that mother’s previous antisocial behavior places children at higher risk for SUI (no antisocial behavior: 18.1 % of the children have a SUI; more than two antisocial behaviors, 27.4 % of the children have a SUI). Boys are more at risk for SUI than girls (23.8 % vs. 8.1 % respectively). Children with frequent hyperactive behaviors also have a higher likelihood of being involved in a SUI.

For RUI, three pregnancy risk factors were significantly related to the occurrence of repeated injuries in children between birth and 42 months of age: maternal smoking, maternal use of medication on prescription, and maternal use of medication without prescription. Children of mothers who smoked, who took medication on prescription and/or without prescription had a higher risk of sustaining RUI than children of mothers who neither smoked nor used medication on prescription and/or without prescription (4,5 % vs 7.2 %; 4.2 % vs 6.5 %, and 4.3 % vs 6.4 %, respectively, all differences *p* < 0.05). Mother’s age at birth of first child was not related to RUI.

Boys (6.8 %) were significantly more at risk for RUI than girls (3.5 %; *p* < 0.05) and children of single mothers also had a higher risk of sustaining RUI (9.7 % vs 4.9 %; *p* < 0.05). Mother’s educational level, her report of antisocial behavior during adolescence, use of alcohol and illicit substances, family income and depressive symptoms were unrelated to RUI. As to the children’s characteristics, their BMI and hyperactive behaviors at age 17 months were not associated with RUI. However, children with a difficult temperament were more often involved in RUI (Tables 1 and 2). Finally, there was a tendency for mothers of children with RUI to feel less efficient as a mother, to perceive less impact on their child’s behavior, and to use a more coercive parenting style (Table 2).

**Table 2:** Sample characteristics: child temperament and hyperactivity and socialization, means and standard deviations for the children with or without single unintentional injuries (SI) (*N* = 1678) and repeated unintentional injuries (RUI) (*N* = 1770)

	SUI		RUI	
	None (SD)	SUI (SD)	None (SD)	RUI (SD)
Child difficult temperament †	2.7 (1.6)	2.7 (1.7)	2.7 (1.7)	3.1 (1.7)
Child hyperactive behavior *	3.4 (2.1)	3.7 (2.3)	3.5 (2.2)	3.8 (2.1)
Maternal depression	1.4 (1.3)	1.4 (1.3)	1.4 (1.3)	1.6 (1.4)
Mean maternal feeling of self-efficacy ^a^	8.5 (1.2)	8.5 (1.3)	8.5 (1.2)	8.3 (1.4)
Mean maternal perception lack of a impact ^a^	8.4 (1.8)	8.4 (1.8)	8.4 (1.8)	8.8 (1.3)
Mean mother’s tendency to coercion ^a^	3.3 (2.4)	3.5 (2.5)	3.4 (2.4)	3.8 (2.3)

*SUI:* *Significant: *p* < 0.05;

*RUI:* †Significant: *p* < 0.05; ††Significant: *p* < 0.01; ^a^Significant: 0.05 < *p* < 0.10

Results from multivariate logistic regression analyses indicate that the likelihood of a SUI increases when mothers were involved in two or more forms of antisocial behavior during adolescence (*OR* = 1.72), when they were age 20 or older at first birth (age 20–23: *OR* = 1.79; 24 and older: *OR* = 1.82), and when the was child a male (*OR* = 1.36) and manifested a higher level of hyperactive behaviors (*OR* = 1.06) (Table 3).

For RUI, the final model largely confirmed the bivariate analysis. The three forms of substance use were significant predictors of RUI: smoking (*OR* = 1.7), medication on prescription (*OR* = 1.5) and medication without prescription (*OR* = 1.5). Two child characteristics were significant predictors of RUI: sex and child temperament. Boys (*OR* = 2) and children with a difficult temperament (OR = 1.1) had significantly higher rates of RUI. Mothers who were single at the child’s birth (OR = 2) and mothers who did not perceive themselves as having an impact on their child’s behavior (OR = 1.1) also had children at higher risk of RUI. There was a tendency for maternal feelings of self-efficacy to predict RUI with mothers feeling less efficient as a parent tending to predict higher RUI rates (*OR* = 0.87; *p* = 0.08) (Table 4).

**Table 3:** Logistic regression for predictors of single unintentional injuries (SUI) (5 to 42 months) (*N* = 1678)

Characteristics		OR Significance	95 conf. interval
			Lower–Upper
Mother’s antisocial behavior (prior to end of high school)	None		
	One	1.24	0.95–1.63
	Two or more	1.72**	1.25–2.36
Mother’s age at first birth	20 or younger		
	20–23	1.79*	1.07–3.00
	24 and older	1.82*	1.12–2.94
Child’s sex		1.36**	1.07–1.73
Child hyperactive behavior		1.06*	1.00–1.12
Constant = −1.63**			
2LL = 1692.7; Cox & Snell R2 = 0.02; Nagelkerke R2 = 0.03			

*Significant: *p* < 0.05; **Significant: *p* < 0.01

^a^ *p* = 0.08

**Table 4:** Logistic regression for predictors of repeated unintentional injuries (RUI) (5 to 42 months) (*N* = 1770)

Characteristics	OR Significance	95 conf. interval
		Lower–Upper
Prenatal smoking	1.68*	1.06–2.65
Prenatal medication on prescription	1.53*	0.99–2.34
Prenatal medication without prescription	1.54*	1.00–2.38
Sex	2.01**	1.29–3.15
Mother single at child’s birth	2.05*	1.00–4.20
Temperament	1.13*	0.99–1.29
Mean maternal feeling of self-efficacy (17 months)	.87^a^	0.73–1.02
Mean maternal perception of impact (17 months)	1.15*	0.99–1.32
Constant = −2.9**		
2LL = 680.2; Cox & Snell R2 = 0.02; Nagelkerke R2 = 0.06		

*Significant: *p* < 0.05; **Significant: *p* < 0.01

^a^ *p* = 0.08

## Discussion

The aim of the present study was first, to prospectively examine the development of unintentional injuries from birth to 42 months of age, and, second, whether SUI and RUI could be independently predicted from a multitude of factors such as prenatal maternal characteristics and behaviors during pregnancy and maternal and child characteristics measured during the first 17 months of life. The results show that close to one in five of the children had sustained one unintentional injury by the age of 42 months. About 1 in 20 of the children had been involved in two or more unintentional injuries. The latter group was responsible for more than a third of all reported injuries from birth to 42 months of age. This group of children with RUI is particularly vulnerable and therefore should be a priority for preventive action.

The most surprising finding of the present study was the contrast between the findings for SUI and RUI. The results support a life-course perspective of unintentional injuries for both outcomes; however, the specific predictors for SUI and RUI differ.

The findings for SUI show that mother’s early antisocial behaviors as well as her age at first birth are significant predictors. Children of mothers involved in antisocial behavior as well as those of older mothers at first birth have a higher likelihood of SUI. Certain child characteristics are important as well: Boys and children with higher levels of hyperactive behavior are at increased risk of SUI.

The results regarding RUI highlight the importance of the prenatal phase: prenatal smoking, and medication use (without or on prescription) predicted future RUI. In addition, the child’s sex and difficult temperament, and being a single parent were significant risk factors for RUI. Parenting behavior, namely maternal perception of impact and maternal feelings of self-efficacy (at the verge of statistical significance) as measured at the age of 17 months, were also associated with RUI.

The main finding reveals that maternal smoking during pregnancy, prenatal use of medication on prescription and medication without prescription were related to RUI but not to SUI during the first years of the child’s life. To the best of our knowledge, only one previous study, namely Ellwood et al. (1993), reported that young children whose mothers were substance users during pregnancy had more hospitalizations for injuries and poisonings than children of a control group. In that study substance use was defined as alcohol use and/or illegal substance use. Ellwood et al. (1993) used the ICD-9-CM (International Classification of Diseases, Ninth Revision, Clinical Modification) Diagnosis Codes and the Short Doyle Service Codes for the Identification of Substance Exposed Children which also included “other type of drug dependence” (Ellwood et al. 1993, p. 5). Tobacco smoking was not included in their study. Sun and colleagues found that low gestational age and low birth weight were related to higher rates of unintentional injuries. Because prenatal smoking is associated with low birth weight (Gray et al. 2004), both Sun and colleagues’ study and the present study point to the possibility of biological programming having an impact on children’s unintentional injuries, as proposed by Wadsworth (1998). However, the causal process remains unknown at the moment.

A potential pathway is that mother’s prenatal smoking and medication (on or without prescription) as well as low gestational age and low birth weight are risk factors for behavior problems in children (Linnet et al. 2003; Rice and Barone 2000), which, in turn lead to risky behavior and unintentional injuries (Rowe et al. 2004; Schwebel et al. 2004). However, child behavior was taken into account in this study, namely temperament at age 5 months and hyperactive behavior at age 17 months. Therefore, the present study does not support this pathway.

It is also unlikely that the relationship between children’s repeated unintentional injuries and maternal smoking and medication use (without or on prescription) could be explained by maternal depression (Schwebel and Brezausek 2008), because maternal depressive symptoms were related to neither SUI nor RUI in the present study.

Although some studies reported that prenatal alcohol use predicts poor emotional control and behavior problems in children (Dixon et al. 2007), we did not find any association between alcohol and illegal substance use and SUI or RUI. An explanation might come from a study by Brooke et al. (1989). They reported that prenatal smoking was more important than alcohol use as prenatal smoking led to a 5 % reduction in birth weight while alcohol had an effect on birth weight only in smokers (Brooke et al. 1989). However, it should be noted that we had only limited information on alcohol use and missed information on drinking to intoxication.

Children of mothers who were single at the child’s birth were at higher risk of RUI but not of SUI. The latter finding replicates O’Connor et al. (2000).

Boys were more at risk for SUI and RUI than girls. This overrepresentation of boys is an almost universal finding (Owens 2002).

Child temperament at age 5 months was related to later RUI but not to SUI. Previous research reported that aspects of difficult temperament such as extraversion, low inhibitory control and low soothability increase the risk of unintentional injuries (Schwebel and Brezausek 2004; Van Aken et al. 2007). In contrast, children’s hyperactive behavior was related to SUI but not to RUI in the present study. The literature on hyperactivity has not been conclusive. Similar to the present study, some studies found no relationship between hyperactive behavior and unintentional injuries (Schwebel et al. 2002) but others reported that hyperactivity was linked to increases in unintentional injuries (Schwebel et al. 2002; Stoddard and Saxe 2001).

The present study found no relationship between socio-economic variables and SUI or RUI. These findings are in line with the literature (Petridou and Belechri 2002).

Mother’s depressive symptoms were unrelated to both SUI and RUI. This is at odds with previous research on maternal depression (Schwebel and Brezausek 2008), although some studies, like the present one, did not find a relationship between maternal depression and children’s injuries (Johnston and Martin-Herz 2010).

Maternal antisocial behavior during high school was associated with SUI but not with RUI. In the case of SUI, this confirms previous research (Hjern and Bremberg 2002).

To our surprise, older mothers reported more SUI but not RUI. This is an unexpected finding, as several studies showed that children of young mothers have relatively high rates of unintentional injuries (Ekeus et al. 2004). A possible explanation of this unexpected finding is that adolescent mothers are afraid authorities might take their child away, and, for that reason, are reluctant to admit that their child suffered an unintentional injury. (Bennett Murphy et al. 2001, p. 305) reported that “adolescent mothers themselves often expressed the concern that if they described the injuries in their children, they would somehow be blamed.” This might lead to underreporting their children’s accidents in surveys.

Among the predictors measured at age 17 months, the child’s BMI did not increase the risk of single or repeated injuries. Although one study based on a relatively small sample (*N* = 79) reported that low BMI increases the likelihood of injuries (Thompson et al. 2011), the present research could not confirm this finding.

Parental perceptions and behaviors were unrelated to SUI but were significantly associated with RUI. Maternal perceptions of lack of impact and of low self-efficacy increased the likelihood of RUI. However, coercive parenting was unrelated to RUI. In other words, when mothers felt they have little impact on their child’s development and reported feeling less able to accomplish tasks related to parenting, the likelihood of RUI increased. These findings are in agreement with studies that showed that parental behaviors, specifically the lack of adequate supervision, increases the likelihood of unintentional injuries (Morrongiello 2005).

How can we interpret the differences we found with respect to the predictors of SUI and RUI? The present findings for SUI are more in line with the research literature than the results for RUI. As single injuries are more prevalent than repeated ones, most findings in previous research is based more heavily on SUI and, therefore, largely confirm our findings with respect to SUI. In contrast, few studies investigated RUI and those who did, with the exception of one (Ellwood et al. 1993), did not examine the effects of substance use. The present study adds to the literature by showing the importance of distinguishing between SUI and RUI and by showing the importance of prenatal substance use for RUI.

It is important to mention the limitations of this study. First, mothers provided the information on both the risk factors and their children’s unintentional injuries. Self-report information is vulnerable to biases linked to the characteristics of the interviewees. However, in general self-reported information on substance use has been found reasonably reliable (Jacobson et al. 2002; Lawrence et al. 2003). Mothers may also forget about the unintentional injuries which their child sustained. However, the effect of forgetting unintentional injuries may be limited as we summed the answers of four times of measurement which had intervals of only 12 months. Also, only one person provided information. As this was usually the mother, information from the other parent, usually the father, is missing. Also, it was unknown whether the child’s injuries occurred specifically when the child was under the responding parent’s direct supervision.

Second, it is difficult to ascertain that the reported injuries were unintentional (Hoyert et al. 2000; McDorman et al. 2002). It is therefore possible that some of the reported unintentional injuries were actually intentional.

Third, it is possible that unmeasured variables during pregnancy and in the first years of life would explain the risk factors we have identified. For instance, no information was available about assistance with caregiving and whether parents felt they needed or wanted additional assistance. We also did not have any information on the number of children in the household, or whether injuries occurred inside or outside the home, whether they occurred in the parent’s custody, during day care or while the child was in someone else’s custody.

Future research should include additional parental factors such as their health status and work obligations. More generally, future research needs to replicate our findings with repeated injuries, but also further investigate single vs. multiple injuries by including an injury severity scale.

To the best of our knowledge, this is the first study to investigate the development and predictors of RUI in a representative sample of young children during the first 4 years of life. More specifically, this study examines whether prenatal risk factors and factors measured in the first 17 months are independent predictors of children’s RUI during the first 42 months of life. In addition, RUI are measured by summing up information provided at four different waves of data collection.

The present findings have implications for policymakers and practitioners for the prevention of costly unintentional injuries during early childhood and possibly during later developmental stages. First, prevention of smoking and certain forms of medication use during pregnancy could contribute to prevent unintentional injuries. A recent review concluded that programs for smoking cessation can help women to quit smoking during pregnancy (Lumley et al. 2009). However, the overall effect was small as - on average - only 6 % of the women stopped smoking.

Second, interventions for prenatal and postnatal support to women who cumulate the risk factors we have identified (smoking, use of medication on or without prescription) should help prevent numerous child development problems which include unintentional injuries and behavior problems. A meta-analysis by Kendrick (Kendrick et al. 2007) showed that pre- and postnatal parenting interventions can prevent unintentional injury rates. Generally these interventions are part of a multifaceted intervention with a parenting component that also includes child safety. An example of an intervention addressing prenatal as well as postnatal risk factors is the Nurse-Family Partnership program that was studied in Elmira, Denver and Memphis. In all settings the intervention helped pregnant women to reduce prenatal smoking (Kitzman et al. 1997; Olds et al. 1986a, b, 2002). For two sites (Elmira and Memphis) information on child unintentional injuries was available and reductions in unintentional injuries were reported (Kitzman et al. 1997; Olds et al. 1986a, b, 1994). A comprehensive and multimodal approach seems to be necessary to tackle the multiple and developmentally intertwined risk factors associated with unintentional repeated injuries among children.

In conclusion, this study suggests that professionals working with pregnant women need to pay special attention to the lifestyle behaviors of these future mothers such as smoking and use of medication. These behaviors may put their children at increased risk of unintentional injuries. In addition, professionals should continue to provide support to families after the birth of the child to counter the effects of further risk factors for unintentional injury such as the gender of the child and difficult temperament.
